# Supplementary material for: Engraftment of Allotransplanted Tumor Cells in Adult rag2 Mutant Xenopus tropicalis
Source: Cancers (Basel). 2022 Sep 20;14(19):4560. doi: 10.3390/cancers14194560 (PMC9559464; doi:10.3390/cancers14194560)
Supplement: Supplementary file 1 [file cancers-14-04560-s001.zip › cancers-1919726-supplementary.pdf]

Supplementary Materials: Engraftment of Allotransplanted Tumor Cells in Adult *rag2* Mutant *Xenopus tropicalis.*

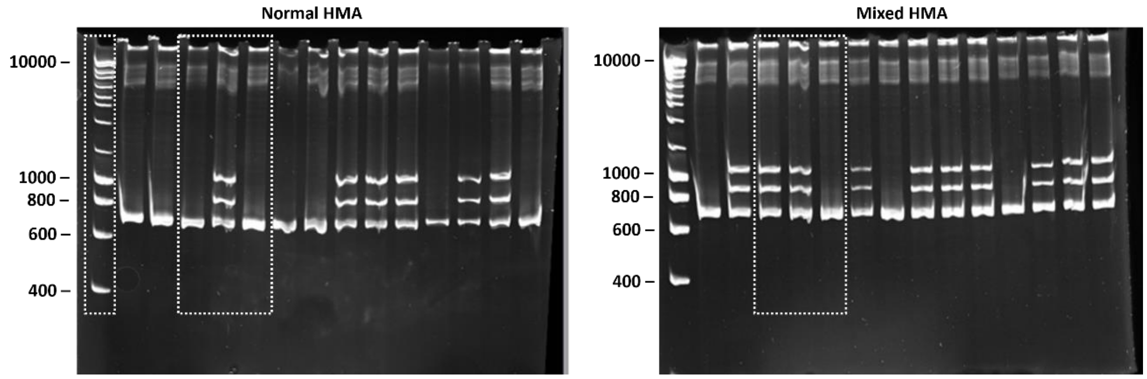

Figure S1. Uncropped blots from Figure 1C. *rag2* gene genotyping of sampled F_2 animals. Images taken from DNA electrophoresis gels after performing a normal HMA (left) and mixed HMA (right). Multiple bands present in both gels indicate heterozygous animals, while extra bands only appearing after performing the mixed HMA (right gel) relate to homozygous mutant animals. Absence of any extra bands is indicative of wild type animals. Dashed white line boxes highlight cropped lanes shown in Figure 1C, other lanes represent littermate animals subjected to the genotyping analysis.

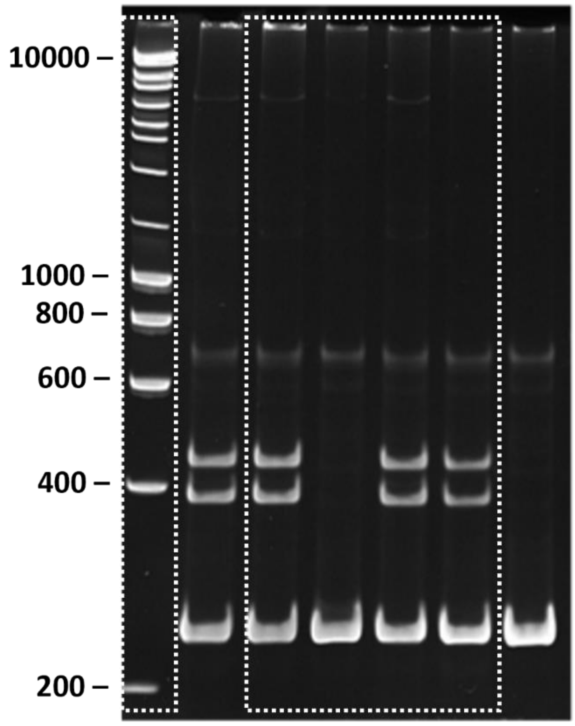

Figure S2. Uncropped blot from Figure 2F. Mixed HMA analysis for the *tp53* gene. Dashed white line boxes highlight cropped lanes shown in Figure 2F. From left to right: tumor sample 1 (*tp53*$^{-/-}$ donor animal), tumor sample 2 (*tp53*$^{-/-}$ donor animal), liver of transplanted *rag2*$^{-/-}$ animal (without grafts), two tumor grafts obtained from the transplanted *rag2*$^{-/-}$ animal and DNA from a not injected wild type animal.
